# Ustekinumab Improves Psoriasis without Altering T Cell Cytokine Production, Differentiation, and T Cell Receptor Repertoire Diversity

**DOI:** 10.1371/journal.pone.0051819

**Published:** 2012-12-14

**Authors:** Kenshiro Tsuda, Keiichi Yamanaka, Makoto Kondo, Kimiko Matsubara, Ryogen Sasaki, Hidekazu Tomimoto, Esteban C. Gabazza, Hitoshi Mizutani

**Affiliations:** 1 Department of Dermatology, Mie University, Graduate School of Medicine, Tsu, Mie, Japan; 2 Department of Neurology, Mie University, Graduate School of Medicine, Tsu, Mie, Japan; 3 Department of Immunology, Mie University, Graduate School of Medicine, Tsu, Mie, Japan; University Medical Center Freiburg, Germany

## Abstract

Ustekinumab is a fully human IgG1κ monoclonal antibody targeting interleukin (IL)-12/23 p40 subunit. The role of IL-12/23-mediated pathway in the mechanism of various inflammatory disorders especially psoriasis has been well recognized. Recently the long-term efficacy and safety of ustekinumab in patients with moderate-to-severe psoriasis has been evaluated in phase 2/3 clinical trials, and the results showed no significant risk for serious adverse effects, infections, or malignancies. Ustekinumab inhibits the function of the IL-12/23 p40 subunit, and therefore it is believed that inhibition of IL-12 p40 pathway decreases IFN-γ production. The major concern for the use of ustekinumab is the possibility of increased immunosuppression due to low IFN-γ production. However, the effects of ustekinumab on CD4^+^ T cell function have not been fully investigated so far. In this study, we explored changes in cytokine production by memory CD4^+^ T cells as well as in the differentiation of naïve T cells to helper T cell (Th) 1, Th2, or Th17 cells in psoriasis patients treated with ustekinumab. The effect of the treatment on T cell receptor repertoire diversity was also evaluated. The results showed that ustekinumab improves clinical manifestation in patients with psoriasis without affecting cytokine production in memory T cells, T cell maturation, or T cell receptor repertoire diversity. Although the number of patients is limited, the present study suggests that T cell immune response remains unaffected in psoriasis patients treated with ustekinumab.

## Introduction

Psoriasis is a chronic immune-mediated skin disorder with frequent clinical relapse [1]. The majority of patients with moderate-to-severe psoriasis require specific topical and systemic therapies including phototherapy (psoralen ultraviolet A therapy (PUVA) or narrow-band ultraviolet B (NB-UVB)), methotrexate [2], cyclosporine [2], and retinoids [3]. However, long-term follow-up during these therapies is generally difficult because of cytotoxicity-related adverse effects, treatment failure, or patient dissatisfaction [4], [5].

Recently, several biologic agents (biologics) have been reported for the treatment of psoriasis [6]–[8]. Biologics have high target specificity and their use is associated with limited organ toxicity. However, the risk of cancer or infection during long-term use in patients with psoriasis has not been as yet investigated.

IL-12 and IL-23 play important roles in the pathogenesis of psoriasis [9]. In psoriasis patients, IL-12 and IL-23 are involved in immune response mediated by helper Th1 [10] and Th17 [11], [12]. IL-12 and IL-23 are heterodimers with a common p40 subunit. The binding of the subunits to their respective receptors activates specific intracellular signaling pathways [13], [14]. Ustekinumab (Stelara®; Janssen Biotech, Inc., Horsham, PA), a fully human IgG1κ monoclonal antibody, binds to the common p40 subunit of IL-12 and IL-23, and blocks activation of the receptors of these cytokines in dendritic cells and monocytes. Recent studies have shown significant effectiveness and safety of ustekinumab in moderate-to-severe plaque-type psoriasis during phase 2 [15] and phase 3 clinical trials [16]–[19]. However, IL-12 is known to have anti-cancer activity by promoting IFN-γ production, therefore there is risk of cancer development due to immunosuppression. The effects of ustekinumab on the production of IL-12/IL-23 are known but its effects on T cell function are not completely understood.

In the present study, we investigated the influence of ustekinumab on T cell cytokine production, differentiation of naïve T cells and on the T cell receptor repertoire diversity in psoriasis patients.

## Materials and Methods

### Subjects

Five psoriasis patients and five healthy volunteers were enrolled in this study. Patients with psoriasis eligible for the use of biologics were included in the study. Briefly, they fulfilled the rule of 10: Psoriasis Area and Severity Index (PASI)≧10, and/or Body Surface Area (BSA)≧10%, and/or Dermatology Life Quality Index (DLQI)≧10. The phonotypical character and response to the biologics are shown in table 1.

**Table 1:** Background of five patients and five healthy controls.

	patient1	patient 2	patient 3	patient 4	patient 5	control 1	Control 2	control 3	control 4	control 5
Disease duration	12 years	16 years	10 years	1 year	14 years	none	none	none	none	none
PASI score										
Pre therapy	16.8	13.2	17.2	49.2	7.3	0	0	0	0	0
Post 1^st^ infusion	3.8	4.4	4.8	4.2	6.1	0	0	0	0	0
Post 2^nd^ infusion	5.7	2.0	2.0	0.9	14.7	0	0	0	0	0
WBC (before)	6930	6530	5200	8150	6070	4600	6340	7200	5830	8280
Lymphocyte(%)	28.9	26.5	24.7	13.7	32.3	28.5	33.0	27.7	32.8	37.4
WBC (Post 1)	6850	7080	5360	7160	8560	5010	6020	7080	5920	8100
Lymphocyte(%)	27.4	32.5	34.3	15.5	22.5	30.1	30.3	29.1	31.2	36.2
WBC (Post 2)	7120	6500	5230	6700	6750	4670	6730	6830	6310	8310
Lymphocyte(%)	31.3	38.3	26.4	13.9	20.6	29.2	29.1	30.6	28.5	36.4

The PASI score of the patients was high before ustekinumab therapy, and improved dramatically after the treatment. However, the PASI score of case 5 was increased at one month after the third therapy. WBC counts and the ratio of lymphocytes in all patients and controls were preserved during all the course of the study.

### Psoriasis Treatment Protocol and Blood Sampling Schedule

Ustekinumab was administrated on weeks 0, 4, and 12. In principle, ustekinumab at a dose of 45 mg was administered intradermally during each therapy. Blood was sampled one month after the third administration after obtaining written informed consent from the subjects. Blood sampling was performed three times in two psoriasis patients and in one healthy volunteer (before the first administration, and one month after the second and third administration). The investigational protocol was approved by the Institutional Review Board (IRB) of Mie University Hospital (Permit Number 2096).

### Antibodies and Reagents

Phytohemagglutinin (PHA), Phorbol 12-myristate 13-acetate (PMA), and ionomycin were purchased from Sigma-Aldrich (St. Louis, MO, USA). Purified anti-human CD3 mAb, anti-hCD28 mAb, anti-hCD8a-FITC mAb, anti-hTCR γ/δ-FITC mAb, anti-hIFN-γ-PerCP mAb, anti-hIL-4-PerCP mAb, anti-hIL-17-PerCP mAb, anti-hTNF-α-PerCP mAb, and brefeldin A were purchased from BioLegend (San Diego, CA, USA). Anti-hCD4-FITC mAb, anti-hCD45RA-FITC mAb, anti-hCD3-PerCp mAb, and anti-hCD45RO-PE mAb were purchased from BD/PharMingen (San Diego, CA, USA). Foxp3-PECy5 mAb, and anti-hCD127-FITC mAb were from eBioscience (San Diego, CA, USA), and FITC/PE-human TCR BV antibodies were from Beckman Coulter (Brea, CA, USA). Anti-hIL-4 mAb, anti-hIL-12 mAb, anti- hIFN-γ mAb, and rhIL-12 were purchased from R&D Systems (Minneapolis, MN, USA). Recombinant hIL-1β, rhTGF-β, rhIL-6, and rhIL-2 were purchased from PeproTech (Princeton, NJ, USA). Complete RPMI 1640 medium was made with 10% heat-inactivated fetal bovine serum (FBS, HyClone Laboratories, INC., South Logan, UT, USA), 2.0 mM L-glutamine, 100 U/ml penicillin, and 100 mg/ml streptomycin (Nacalai tesque, Kyoto, JAPAN).

### Purification of CD4^+^T Cells

PBMCs were isolated and prepared as previously described [20]. Briefly, PBMCs were purified from heparinized peripheral venous blood using Ficoll-Hypaque (Sigma-Aldlich, St. Louis, MO) density gradient centrifugation. Purification of CD4^+^ T cells was done by negative selection using the CD4^+^ T Cell Isolation Kit II (Miltenyi Biotec, Bergisch Gladbach, Germany) according to the manufacturer’s instructions. PBMCs were incubated for 10 min with 20 µl of the antibody cocktail mixture followed by 15 min incubation with 20 µl of magnetic beads per 10^7^ cells. Unconjugated CD4^+^ T cells were then isolated from PBMCs by indirect magnetic labeling using MiniMACS separation LS columns. The cell populations were sorted and analyzed by flow cytometry, and the purity of samples being between 96 and 99%.

### Separation of Naïve and Memory CD4^+^T Cells

Naïve CD4^+^ T cells were obtained by negative selection from purified CD4^+^ T cells using the naïve CD4^+^ T Cell Isolation Kit, and memory CD4^+^ T cells were collected by positive selection. In brief, magnetically unlabeled naïve CD4^+^ T cells passed through the column, whereas labeled memory CD4^+^ T cells were trapped and remained in the column and were washed out with buffer. The purity of both cell populations ranged between 94 and 99%.

### Cell Culture of Memory CD4^+^T Cells

Memory CD4^+^ T cells, suspended in complete RPMI 1640 culture medium, were plated into a flat-bottomed 24-well plate at 1×10^6^ cells/well and incubated with PMA (25 ng/mL), ionomycin (1 µg/mL) and brefeldin A (1 µg/mL) and cultured for 24 h at 37°C in an atmosphere of 5% CO_2_.

### Cell Culture of PBMCs

During the third sampling, PBMCs were cultured in complete RPMI 1640 medium in the flat-bottomed 24-well plate at 1×10^6^ cells/well and incubated with PMA (25 ng/mL), ionomycin (1 µg/mL) and brefeldin A (1 µg/mL) for 24 h at 37°C in an atmosphere of 5% CO_2_.

### Generation of Th1 Cells in vitro

Th1 cells were generated by culturing naïve CD4^+^ T cells (1×10^6^/mL) with PHA (1 µg/mL), rhIL-12 (50 ng/mL), and anti-hIL-4 mAb (500 ng/mL) in a flat-bottomed 24-well plate in 1 mL of complete RPMI1640 culture medium at 37°C with 5% CO_2_. The stimulated T cells were collected and washed on day 3 and expanded in the same culture medium with 100 U/mL of rhIL-2 for an additional 3 days. On day 6, the cells were stimulated with PMA (25 ng/mL) and ionomycin (1 µg/mL) in the presence of brefeldin A (1 µg/mL) for 8 h [20], [21].

### Generation of Th2 Cells in vitro

Th2 cells were generated by culturing naïve CD4^+^ T cells (1×10^6^/mL) with PHA (1 µg/mL), rhIL-4 (200 ng/mL), and anti-hIL-12 mAb (10 µg/mL) in a flat-bottomed 24-well plate in a complete RPMI1640 culture medium at 37°C and an atmosphere of 5% CO_2_. The stimulated T cells were washed on day 3 and expanded in the same culture medium with the addition of 100 U/mL of rhIL-2 for an additional 3 days. On day 6, the cells were stimulated with PMA (25 ng/mL), ionomycin (1 µg/mL) and brefeldin A (1 µg/mL). The cells were incubated for additional 8 h [20], [21].

### Generation of Th17 Cells in vitro

Th17 cells were generated by culturing naïve CD4^+^ T cells (1×10^6^/mL) with rhIL-2 (10 U/mL), rhTGF-β (5 ng/mL), rhIL-6 (20 ng/mL), rhIL-1β (10 ng/mL), rhIL-23 (10 ng/mL), anti-hIL-4 mAb (1 µg/mL), anti-hIFN-γ mAb (1 µg/mL), anti-hCD3 mAb (4 µg/mL), and anti-hCD28 mAb (8 µg/mL) in a flat-bottomed 24-well plate in complete RPMI1640 culture medium at 37°C and an atmosphere of 5% CO_2_. On days 3 and 5, the culture plate was centrifuged, and the media was removed and replaced with fresh media containing all cytokines mentioned above, and antibodies. On day 6, the cells were stimulated with PMA (25 ng/mL), ionomycin (1 µg/mL) and brefeldin A (1 µg/mL). The cells were incubated for 8 h at 37°C and an atmosphere of 5% CO_2_ [20], [22].

### Cell Surface and Intracellular Staining of CD4^+^ T Cells

Cultured CD4^+^ T cells were collected and washed twice with PBS containing 1% FBS. Cell surface antigens and intracellular cytokines were stained according to the formal Cell Surface Immunofluorescence Staining Protocol and Intracellular Cytokine Staining Protocol (BioLegend). Briefly, for analyzing cytokine production from memory cells, the cells were firstly stained with anti-hCD4-FITC mAb. To detect cellular differentiation of naïve T cells into cytokine-producing cells, the cells were firstly stained with anti-hCD45RA-FITC and anti-hCD45RO-PE mAbs. After treatment with the fixation and permeabilization wash buffer, the cells were incubated with PerCP-conjugated anti-hIFN-γ, anti-hIL-4, or IL-17A mAbs. Fluorescence profiles were assessed by flow cytometry using FACSCalibur (BD Biosciences, San Jose, CA), and the data were analyzed using Cell Quest Pro software (BD Biosciences).

### Cell Surface and Intracellular Staining of PBMCs

To analyze cytokine production by CD8^+^ T cells, cultured PBMCs were collected and washed twice with PBS containing 1% FBS. The cells were firstly stained with anti-hCD8a-FITC mAb. After treatment with the fixation and permeabilization wash buffer, the cells are incubated with PerCP-conjugated anti-hIFN-γ or anti-hTNF-α mAb. To analyze cytokine production by γ/δ T cells, similar to CD8^+^ T cells, cultured PBMCs were firstly stained with anti-hTCR γ/δ-FITC mAb. After treatment with the fixation and permeabilization wash buffer, the cells are incubated with PerCP-conjugated anti-hIFN-γ, or anti-hIL-17A mAbs. Fluorescence profiles were analyzed by flow cytometry.

### Staining of Naturally Occurring Regulatory T Cells (nTregs)

For identification of nTreg (FoxP3^+^CD127^low^CD25^high^CD4^+^ T cells), magnetically collected CD4^+^ T cells during the third blood sampling were directly stained with monoclonal antibodies to CD127-FITC and CD25-PE in cell surface staining buffer containing 0.1M PBS and 2% FCS (Biowest, Nuaillé, France). Then intracellular staining with Foxp3-PECy5 antibody was performed according to the manufacture’s instruction (eBioscience, San Diego, CA).

### T Cell Receptor Repertoire Diversity

T cell receptor (TCR) repertoire diversity was investigated as reported previously [23]. To analyze the presence or absence of each TCR β-variable (BV) subfamily during the treatment, PBMC during the third collection were stained anti CD3-PerCp and FITC/PE-human TCR BV antibodies for 20 min at room temperature.

### Statistical Analyses

Statistical analysis was performed using the Mann-Whitney test. A *P*-value of less than 0.05 was considered as statistically significant.

## Results

### Clinical Manifestations

The PASI scores of the patients were 16.8 (patient 1), 13.2 (patient 2), 17.2 (patient 3), and 49.2 (patient 4) before ustekinumab therapy, and they improved to 3.8, 4.4, 4.8, and 4.2 at one month after the second therapy, and 5.7, 2.0, 2.0, and 0.9 at one month after the third therapy, respectively. However, the PASI score of case 5 was 7.3 before ustekinumab therapy, improved to 6.1 at one month after the second therapy, but returned to 14.7 at one month after the third therapy. The WBC count and the percentage of lymphocytes in all patients and controls were stable during all the study (Table 1).

### Ustekinumab Induced no Obvious Suppression of Cytokine Production from Memory CD4^+^T Cells

**Figure 1:**
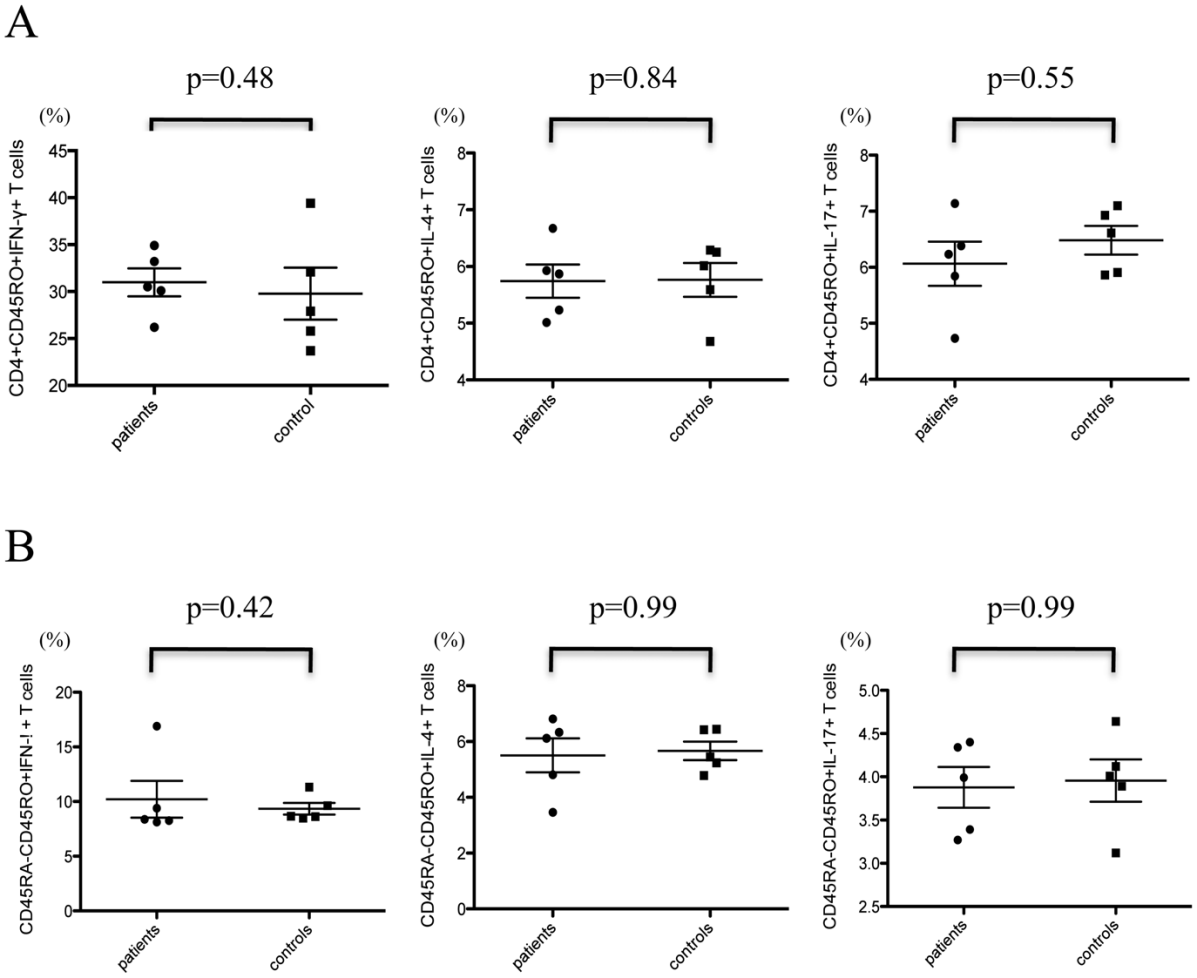
Cytokine production by memory CD4^+^ T cells and differentiation of naïve CD4^+^ T cells to mature cytokine-producing T cells in psoriasis patients during treatment with ustekinumab and in healthy controls. (A) Memory CD4^+^ T cells were stimulated with PMA and ionomycin. Distinct cytokine production by mature cells was observed, and the production of all cytokines was not suppressed in patients with psoriasis treated with ustekinumab. (B) Naïve CD4^+^ T cells were differentiated to mature T cells (Th1/Th2/Th17). Ustekinumab treatment did not change the percentage of cytokine-producing mature T cells compared to the control group.

**Figure 2:**
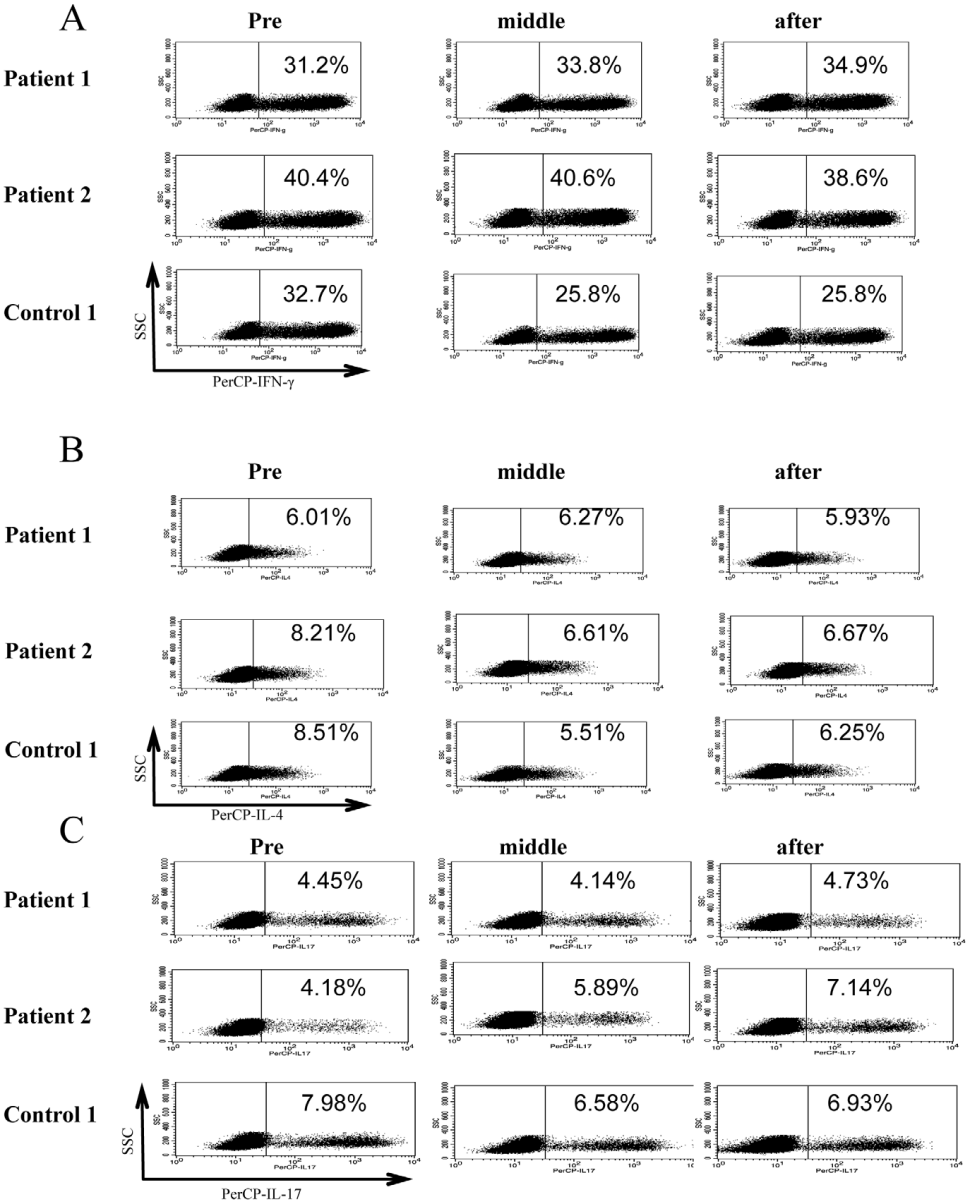
Cytokine production by memory CD4^+^ T cells. Representative flow cytometry data from two patients and control are shown. (A) The percentage of CD4^+^CD45RO^+^IFN-γ^+^ T cells (B) The percentage of CD4^+^CD45RO^+^IL-4^+^ T cells (C) The percentage of CD4^+^CD45RO^+^IL-17^+^ T cells. All cytokine production remained unchanged during treatment.

To determine the effects of ustekinumab on cytokine production from activated memory CD4^+^ T cells, these cells were stimulated with PMA and ionomycin. The results showed distinct cytokine production from mature T cells, and the cytokine production was not significantly suppressed (Fig. 1A). The follow-up data of two patients and one healthy control is shown in Fig. 2; cytokine production was not changed before and after treatment with ustekinumab.

### Ustekinumab Induced no Significant Suppression of Naïve CD4^+^ T Cell Differentiation into Cytokine-producing Mature Cells

**Figure 3:**
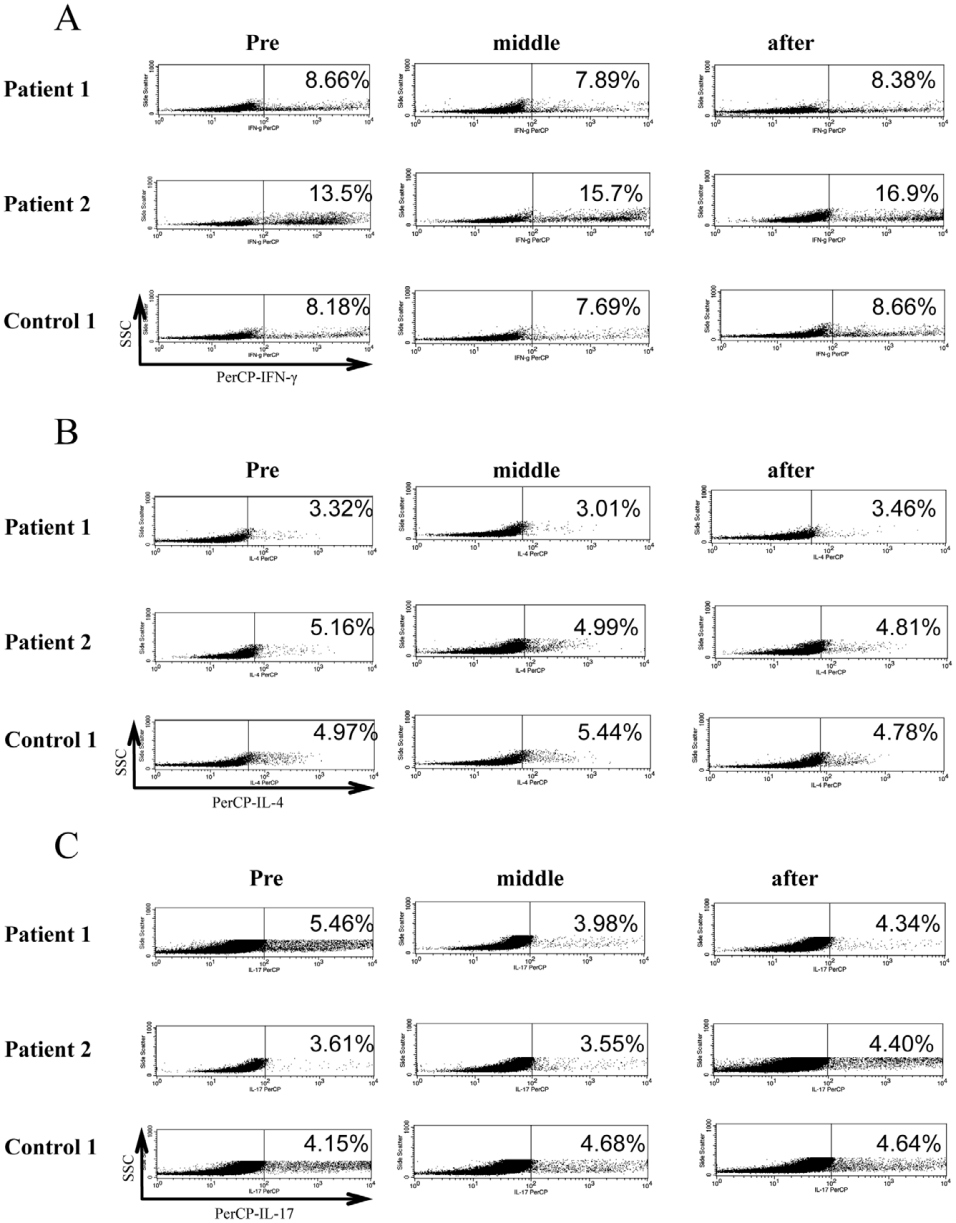
Differentiation of naïve CD4^+^ T cells to cytokine-producing mature cells (Th1/Th2/Th17). Representative flow cytometry data are shown. (A) The percentage of CD45RA^−^CD45RO^+^IFN-γ^+^ cells in the CD4^+^ T cell population (B) The percentage of CD45RA^−^CD45RO^+^IL-4^+^ cells in the CD4^+^ T cell population (C) The percentage of CD45RA^−^CD45RO^+^IL-17^+^ cells in the CD4^+^ T cell population. T cell maturation was not influenced by ustekinumab treatment.

To investigate the effect of ustekinumab on the differentiation of naïve CD4^+^ T cells to Th1, Th2, or Th17 cells, naïve CD4^+^ T cells were cultured in the presence of appropriate cytokines and stimulants as described above. Ustekinumab did not significantly suppress the percentage of CD45RA^−^CD45RO^+^IFN-γ^+^ cells, CD45RA^−^CD45RO^+^IL-4^+^ cells, or CD45RA^−^CD45RO^+^IL-17^+^ cells compared to untreated control group (Fig. 1B). Flow cytometric analysis showed abundant cytokine production from CD45RA^−^CD45RO^+^CD4^+^ T cells. The follow-up study in two patients and one healthy control showed no alteration of T cell maturation by ustekinumab treatment (Fig. 3).

### Naturally Occurring Regulatory T Cells (nTregs)

**Figure 4:**
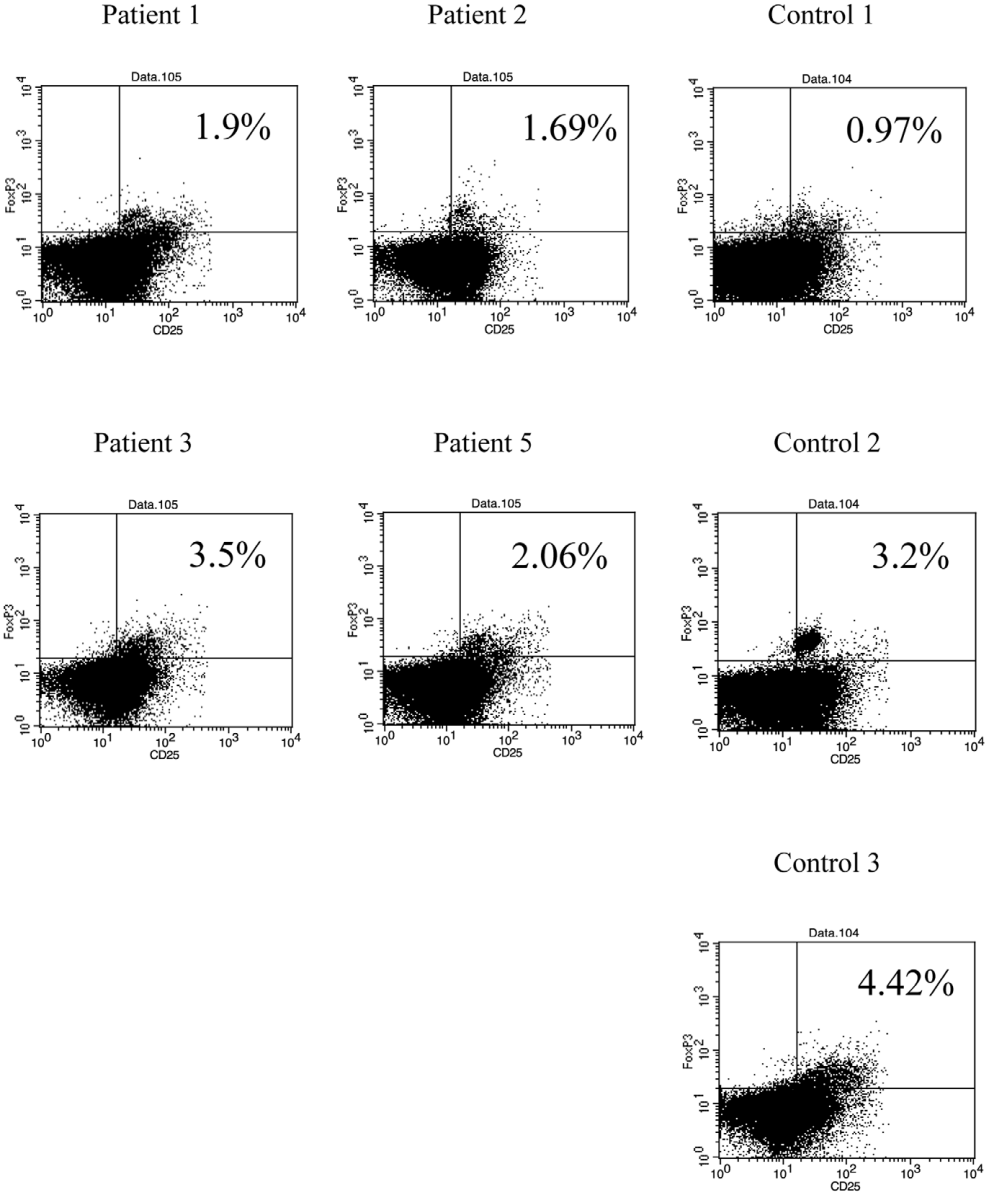
Naturally occurring regulatory T cells. The percentage of nTreg (FoxP3^+^CD127^low^CD25^high^CD4^+^ T cells/CD4^+^ T cells) was similar among the seven volunteers. Flow cytometry data from four patients and three healthy controls are shown.

No significant difference in nTreg (FoxP3^+^CD127^low^CD25^high^CD4^+^ T cells/CD4^+^ T cells) ratio was found among patients and volunteers during the third sampling (Fig. 4).

### T Cell Receptor Repertoire Diversity

**Figure 5:**
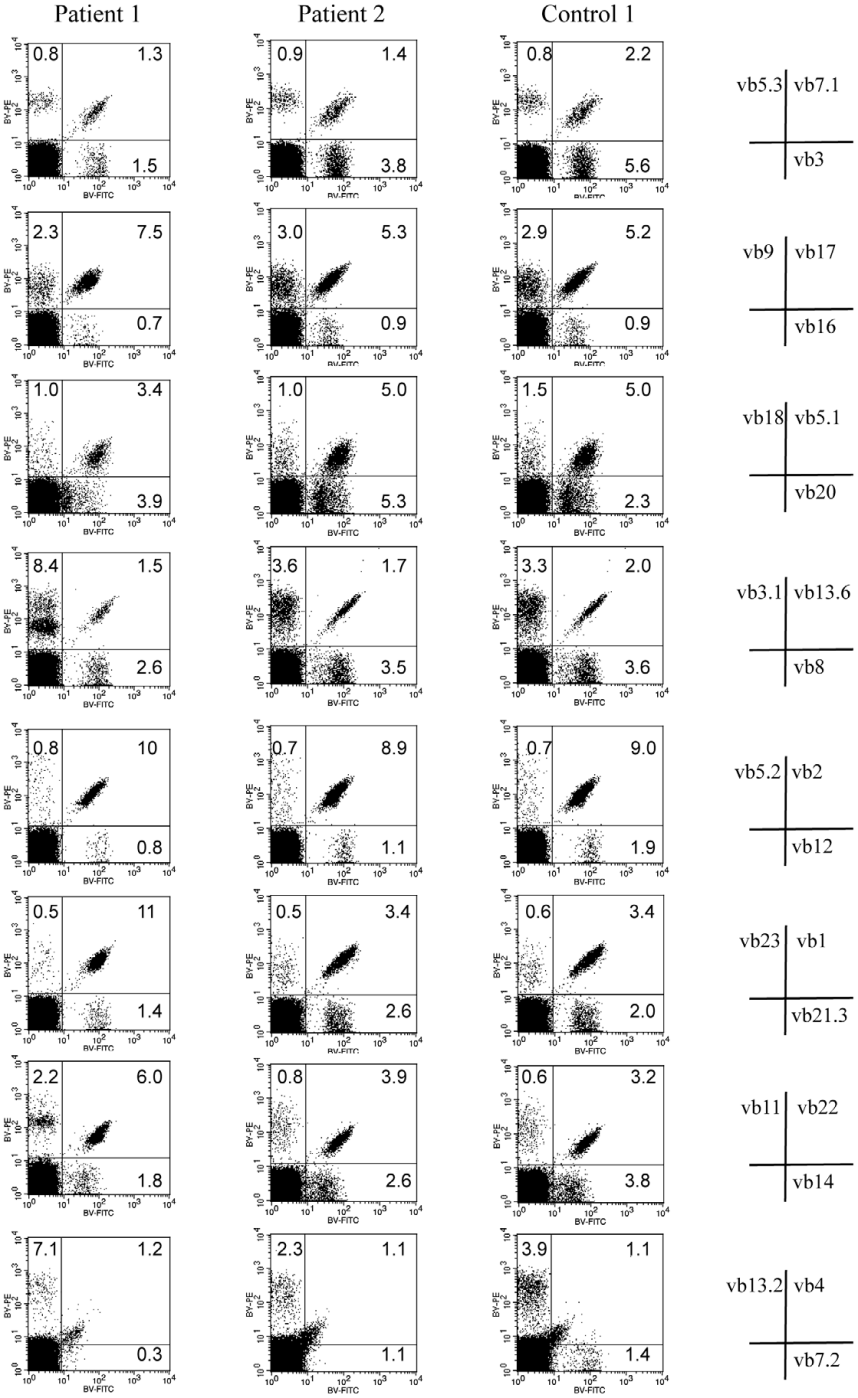
T cell receptor repertoire diversity. TCR BV subfamilies were preserved during treatment with ustekinumab as compared with normal volunteer.

Staining with antibody to TCR BV subfamily showed that all the subfamilies were preserved in the patients compared to normal volunteers, and with no particular collapse in T cell receptor repertoire diversity (Fig. 5).

## Discussion

Recent studies have shown the therapeutic efficacy of several biologic agents for the treatment of refractory psoriasis. Increase in the risk of severe infection, malignancy, or in mortality rate during the use of the agents has not been reported; however, further statistical data are required to confirm the safety of these agents during long-term use [24]. Susceptibility to infection [25] and malignancy [26] has been reported during ustekinumab therapy due to inhibition of IL-12 and IL-23. Recent 2/3 phase clinical trials demonstrated the efficacy and safety of ustekinumab [15]–[19], [27]–[29]. However, there are just a few data on changes in T cell immune response during ustekinumab therapy.

In the present study, we demonstrated cytokine production by Th1 and Th17 cells during ustekinumab therapy, suggesting that blockade of IL-12/IL-23p40 improves skin manifestations without decreasing cytokine production by Th1/Th17cells (Fig. 1A and 2). In addition, TNF-α production from memory CD4^+^ T cells was the same between patients treated with ustekinumab and normal controls (Fig. S1). Furthermore, IL-12/IL-23p40 blockade has limited effects on naïve T cell development with normal T cell development milieu during ustekinumab therapy (Fig. 1B and 3). IL-17 secreted by Th17 cells plays a key role in the inflammatory response in various diseases; the level of IL-17 production and Th17 cell development remained unchanged during the course of treatment. CD4^+^CD127^low^CD25^high^Foxp3^+^ regulatory T cells (nTreg) play critical role in the suppression of excessive inflammatory response in various diseases including psoriasis. nTregs also regulate local and systemic immune response by maintaining the balance among Th1,Th2 and Th17/22 cells. The function of nTreg was also conserved during the course of treatment (Fig. 4). In addition, the cytokine production by CD8^+^ T cells and γ/δ T cells was similar between patients and controls (Fig. S2 and S3).

In the present study, the skin manifestations of the patients markedly improved, despite unaltered cytokine production and T cell differentiation. The cytokine production and differentiation of T cells in response to infections and malignancies were preserved in the peripheral blood. On the other hand, the excessive production of inflammatory cytokines in the skin lesions was controlled during ustekinumab therapy.

Evaluation of the qualitative alteration in T cell immunity during ustekinumab therapy is also important. Clonal expansion or loss of some T cell clones can be associated with risk of malignancy and infection. TCR BV subfamily immune-staining with TCR BV antibodies is a reliable tool for analysis of T cell receptor diversity; collapse and restoration of T cell receptor diversity was reported in CTCL patients in advanced stages of disease [23], [30]. In the present study, no significant alteration in TCR diversity after ustekinumab therapy was observed, suggesting that ustekinumab has no effects on immunological competence (Fig. 5).

In conclusion, the present data showed that ustekinumab improves clinical manifestations in patients with psoriasis without inducing immunosuppression. However, a study with a larger population and longer follow-up should be carried out to confirm these observations.

## Supporting Information

Figure S1 **The percentage of CD4^+^CD45RO^+^TNF-α^+^ T cells.** The ratio of TNF-α producing memory CD4^+^ T cells was not suppressed in patients with psoriasis during ustekinumab treatment as compared to normal controls.
(TIF)

Figure S2 **Cytokine production by memory CD8^+^ T cells.** Flow cytometry data are shown. (A) The percentage of CD8a^+^IFN-γ^+^ T cells (B) The percentage of CD8a^+^TNF-α^+^ T cells. The production of IFN-γ and TNF-α by CD8^+^ T cells was not suppressed in patients with psoriasis treated with ustekinumab.
(TIF)

Figure S3 **Cytokine production by γ/δ T cells.** Flow cytometry data are shown. (A) The percentage of TCR γ/δ^+^IFN-γ^+^ T cells (B) The percentage of TCR γ/δ^+^IL-17^+^ T cells. The production of IFN-γ and IL-17 from γ/δ T cells was not suppressed in patients with psoriasis treated with ustekinumab.
(TIF)
