# Supplementary figures and images for: Ustekinumab Improves Psoriasis without Altering T Cell Cytokine Production, Differentiation, and T Cell Receptor Repertoire Diversity
Source: PLoS One. 2012 Dec 14;7(12):e51819. doi: 10.1371/journal.pone.0051819 (PMC3522598; doi:10.1371/journal.pone.0051819)

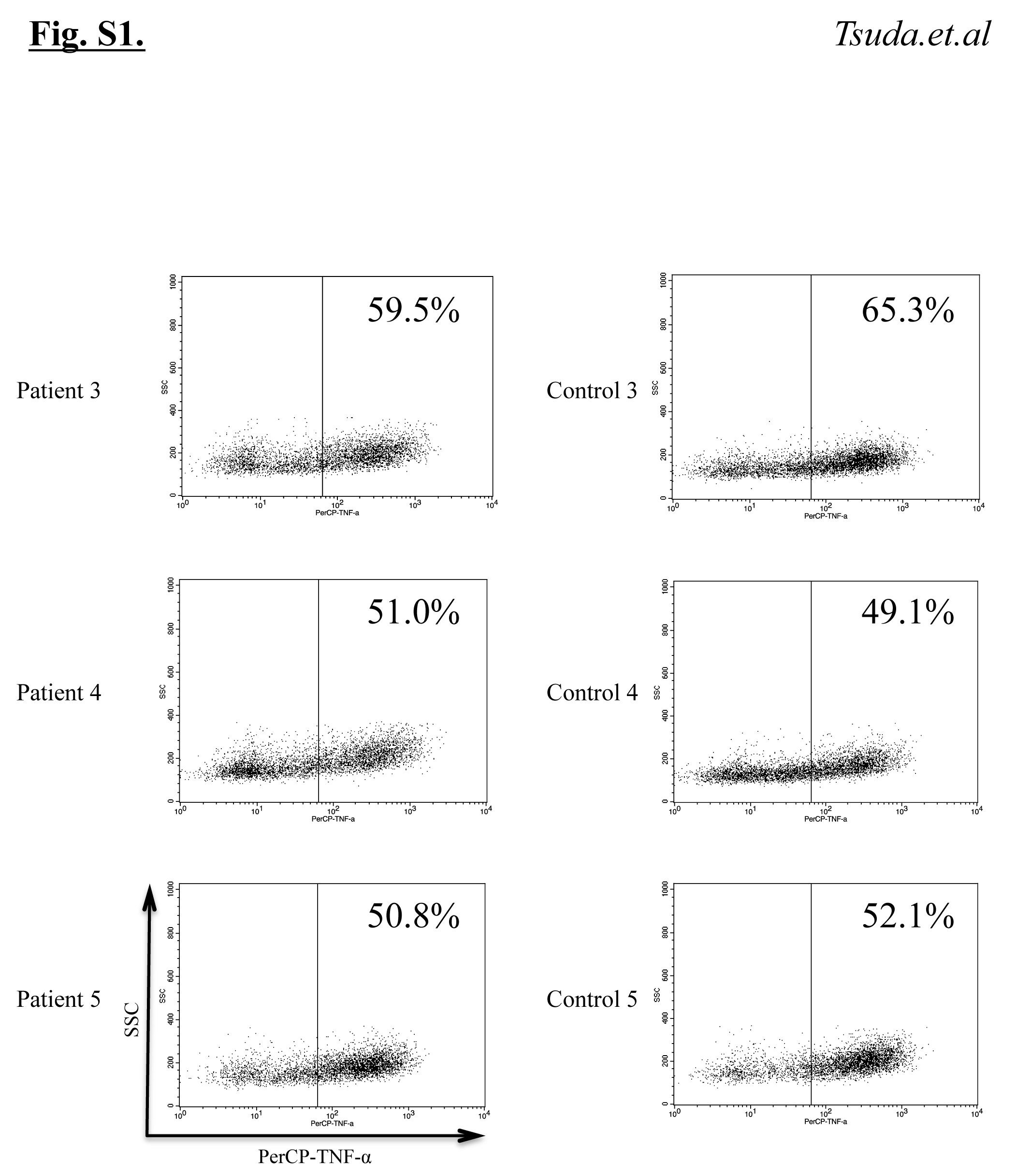

Supplement: Figure S1 — The percentage of CD4+CD45RO+TNF-α+ T cells. The ratio of TNF-α producing memory CD4+ T cells was not suppressed in patients with psoriasis during ustekinumab treatment as compared to normal controls. (TIF) [file pone.0051819.s001.tif]

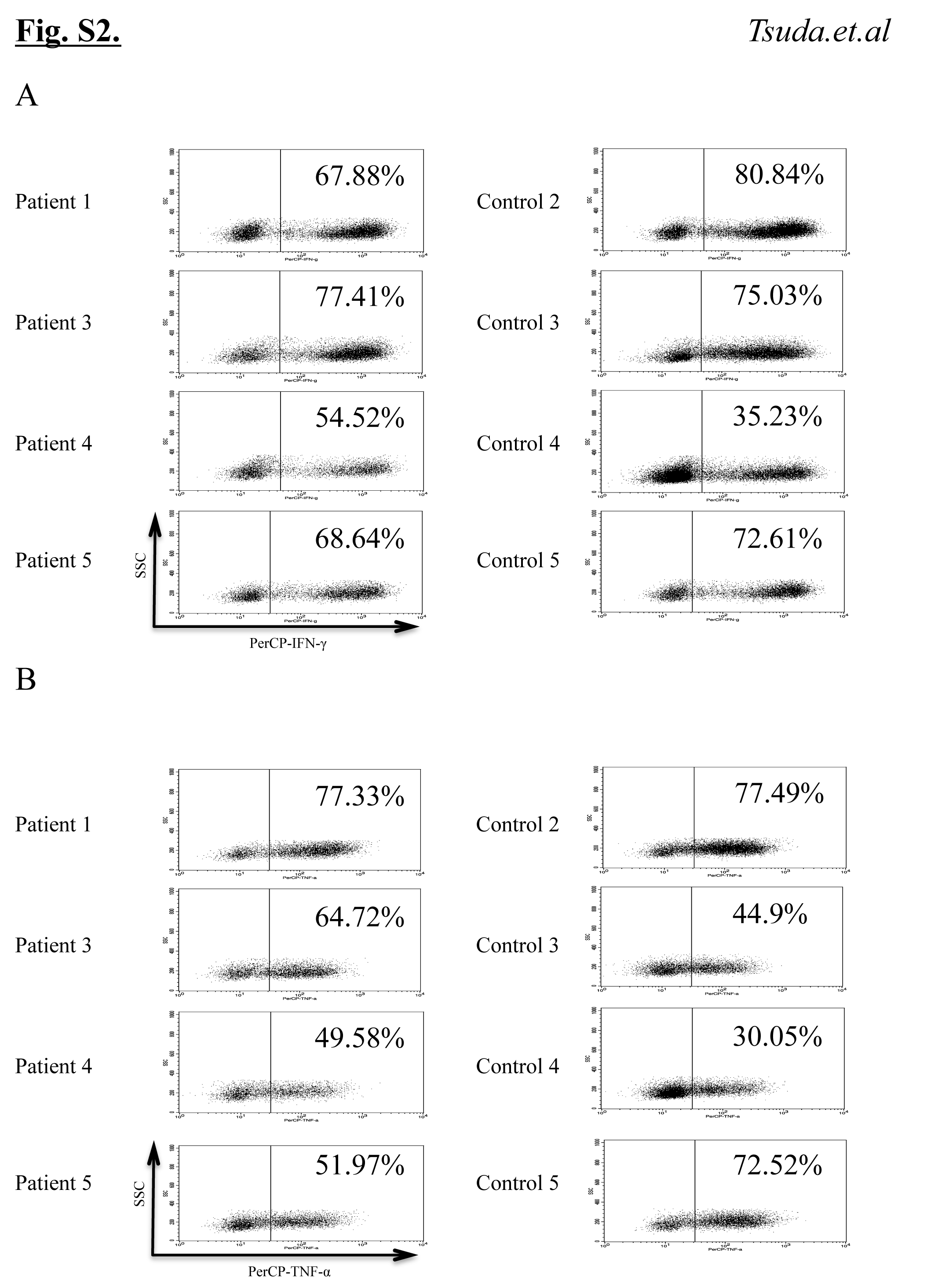

Supplement: Figure S2 — Cytokine production by memory CD8+ T cells. Flow cytometry data are shown. (A) The percentage of CD8a+IFN-γ+ T cells (B) The percentage of CD8a+TNF-α+ T cells. The production of IFN-γ and TNF-α by CD8+ T cells was not suppressed in patients with psoriasis treated with ustekinumab. (TIF) [file pone.0051819.s002.tif]

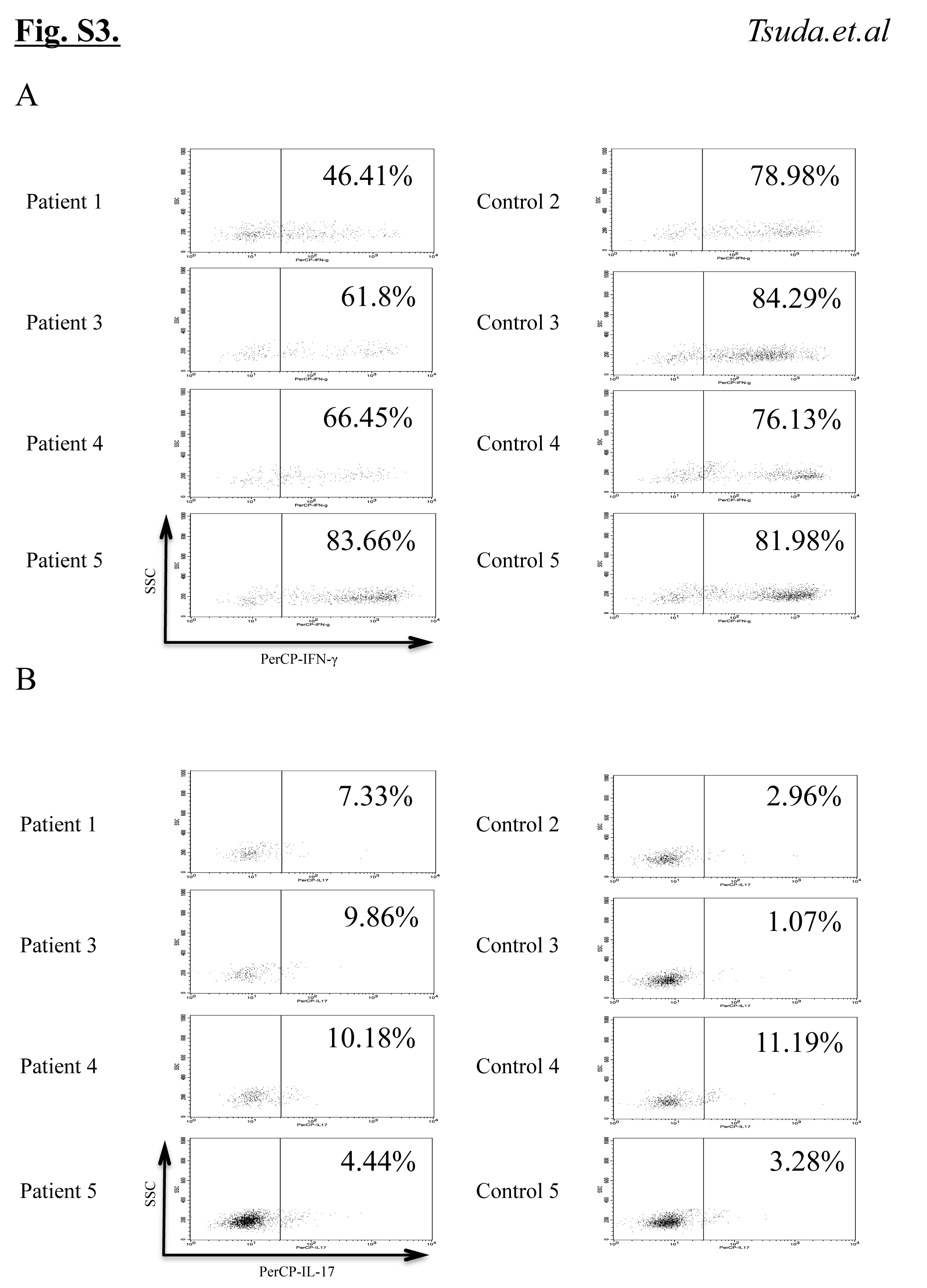

Supplement: Figure S3 — Cytokine production by γ/δ T cells. Flow cytometry data are shown. (A) The percentage of TCR γ/δ+IFN-γ+ T cells (B) The percentage of TCR γ/δ+IL-17+ T cells. The production of IFN-γ and IL-17 from γ/δ T cells was not suppressed in patients with psoriasis treated with ustekinumab. (TIF) [file pone.0051819.s003.tif]
